# Detection of Post-Therapeutic Effects in Breast Carcinoma Using Hard X-Ray Index of Refraction Computed Tomography – A Feasibility Study

**DOI:** 10.1371/journal.pone.0158306

**Published:** 2016-06-30

**Authors:** Susanne Grandl, Anikó Sztrókay-Gaul, Alberto Mittone, Sergey Gasilov, Emmanuel Brun, Alberto Bravin, Doris Mayr, Sigrid D. Auweter, Karin Hellerhoff, Maximilian Reiser, Paola Coan

**Affiliations:** 1 Institute for Clinical Radiology, Ludwig-Maximilians-University Hospital, Munich, Germany; 2 Department of Physics, Ludwig-Maximilians-University, Garching, Germany; 3 ANKA Synchrotron Radiation Facility, Karlsruhe Institute for Technology, Eggenstein, Germany; 4 European Synchrotron Radiation Facility, Grenoble, France; 5 Institute of Pathology, Ludwig-Maximilians-University Hospital Munich, Munich, Germany; University of Notre Dame, UNITED STATES

## Abstract

**Objectives:**

Neoadjuvant chemotherapy is the state-of-the-art treatment in advanced breast cancer. A correct visualization of the post-therapeutic tumor size is of high prognostic relevance. X-ray phase-contrast computed tomography (PC-CT) has been shown to provide improved soft-tissue contrast at a resolution formerly restricted to histopathology, at low doses. This study aimed at assessing ex-vivo the potential use of PC-CT for visualizing the effects of neoadjuvant chemotherapy on breast carcinoma.

**Materials and Methods:**

The analysis was performed on two ex-vivo formalin-fixed mastectomy samples containing an invasive carcinoma removed from two patients treated with neoadjuvant chemotherapy. Images were matched with corresponding histological slices. The visibility of typical post-therapeutic tissue changes was assessed and compared to results obtained with conventional clinical imaging modalities.

**Results:**

PC-CT depicted the different tissue types with an excellent correlation to histopathology. Post-therapeutic tissue changes were correctly visualized and the residual tumor mass could be detected. PC-CT outperformed clinical imaging modalities in the detection of chemotherapy-induced tissue alterations including post-therapeutic tumor size.

**Conclusions:**

PC-CT might become a unique diagnostic tool in the prediction of tumor response to neoadjuvant chemotherapy. PC-CT might be used to assist during histopathological diagnosis, offering a high-resolution and high-contrast virtual histological tool for the accurate delineation of tumor boundaries.

## Introduction

Preoperative neoadjuvant chemotherapy (NAC) followed by surgery is currently the state-of-the-art therapy in locally advanced breast cancer. Additionally, NAC is increasingly applied to patients presenting an early-stage operable breast cancer [1–3]. Due to the preoperative tumor size reduction, NAC can spare a higher percentage of patients from mastectomy since smaller tumors can be treated by breast conserving surgery at survival rates comparable with those achieved by adjuvant chemotherapy [4, 5]. Further advantages of NAC are the possibility of in-vivo monitoring of therapy response and an earlier treatment of possible micrometastases [6].

The pathological response rate of breast carcinomas undergoing NAC is assessed by histopathological analysis of the resected tumor and is an independent predictor for disease-free and overall survival of breast cancer patients compared with non-responders [1–3, 7, 8]. The percentage of pathological complete response (pCR) is approximately 10–20% [9]. However, there is a substantial discrepancy between clinical and pathological response [10–12]. Mammography has a limited sensitivity of 80% in the detection of NAC-induced regressive variations and on the other hand the residual post-therapeutic tumor size is often overestimated by mammography [13, 14]. Approximately 50% of all breast cancers undergoing NAC show regressive tissue changes [5]; however the tumor size does not necessarily change accordingly [15]. Consequently, the definite evaluation of response to NAC relies on the results of postoperative histopathology as the gold-standard, as the clinical X-ray imaging diagnostic tools are not sufficiently sensitive to the small anatomical changes induced by NAS [13–15]. The regressive changes of breast tumors undergoing NAC are evaluated using a scoring system introduced by Sinn et al. [16] or using a very similar scoring system by Chevallier et al. [17].

X-ray phase-contrast imaging (PCI) has emerged within recent years as a new class of techniques for X-ray imaging with improved sensitivity as compared to conventional absorption-based radiography. PCI detects the phase shift that X-rays experience when passing through an object. The combination of X-ray PCI with computed tomography (CT) allows the three dimensional (3D) reconstruction of the refractive index distribution within an object, which can be converted into an accurate 3D map of the sample mass density. Several studies have shown that PCI allows for a superior observation of the breast architecture compared with conventional breast imaging [18–22]. In this study we applied analyzer-based phase-contrast imaging (ABI) technique [21]. This method detects the X-ray phase-shifts induced by the sample with the help of an analyzer crystal, thus enhancing image contrast.

The aims of this study were to 1) demonstrate that 3D index of refraction PC-CT is able to visualize typical changes within breast tumor tissue treated with NAC and 2) show that differential phase-contrast CT techniques are useful for studying the effect of neoadjuvant therapeutics and might have the potential to become a unique and highly accurate diagnostic tool to predict the response rate to NAC and with higher detail than conventional diagnostic techniques.

## Materials and Methods

### Sample Choice

Two formalin-fixated ex-vivo mastectomy specimens (sample A and sample B) from two women suffering from invasive breast cancer who had undergone preoperative NAC were imaged. The breast samples were acquired through the Institute of Gynaecology of the Ludwig-Maximilians-University (LMU), Munich, Germany. The study was conducted in accordance with the Declaration of Helsinki and was approved by the local ethics committee (Ethikkommission of the LMU, Munich, project number 240–10, date of permission 26/08/2010, amendment 30/05/2012). Patients’ written informed consent was obtained before inclusion after adequate explanation of the study protocol. The indication to breast surgery followed recommendations of the interdisciplinary tumor conference by the University of Munich breast center. Inclusion criteria were a histologically proven breast cancer in preoperative core biopsy, with a recommendation to mastectomy, according to gynecological guidelines as well as completed preoperative NAC and completed conventional breast imaging diagnostics (mammography, ultrasonography +/- MRI).

### Preoperative Diagnostics

Preoperative diagnostics included clinical breast examination, clinical standard two-view digital mammography in craniocaudal (CC) and mediolateral-oblique (MLO) projections (Hologic Selenia Dimensions, Bedford, USA) using a standard breast compression paddle, high resolution B-mode ultrasound (standard linear transducer 13.5 MHz, Siemens Acuson Antares, Siemens Healthcare, Germany) and MRI using a dedicated sensitivity-encoding-enabled bilateral breast coil with a 1.5-Tesla system (Magnetom Symphony, Siemens Healthcare, Germany). The clinical diagnostics were perfomed by a specialized radiologist with seven years experience in breast diagnostics. The in-vivo measurements were performed using standard diagnostic software in all modalities (mammography, ultrasound, magnetic resonance imaging) that enables measurements directly on the imaging dataset.

### Sample Preparation for PC-CT Imaging

**Fig 1:**
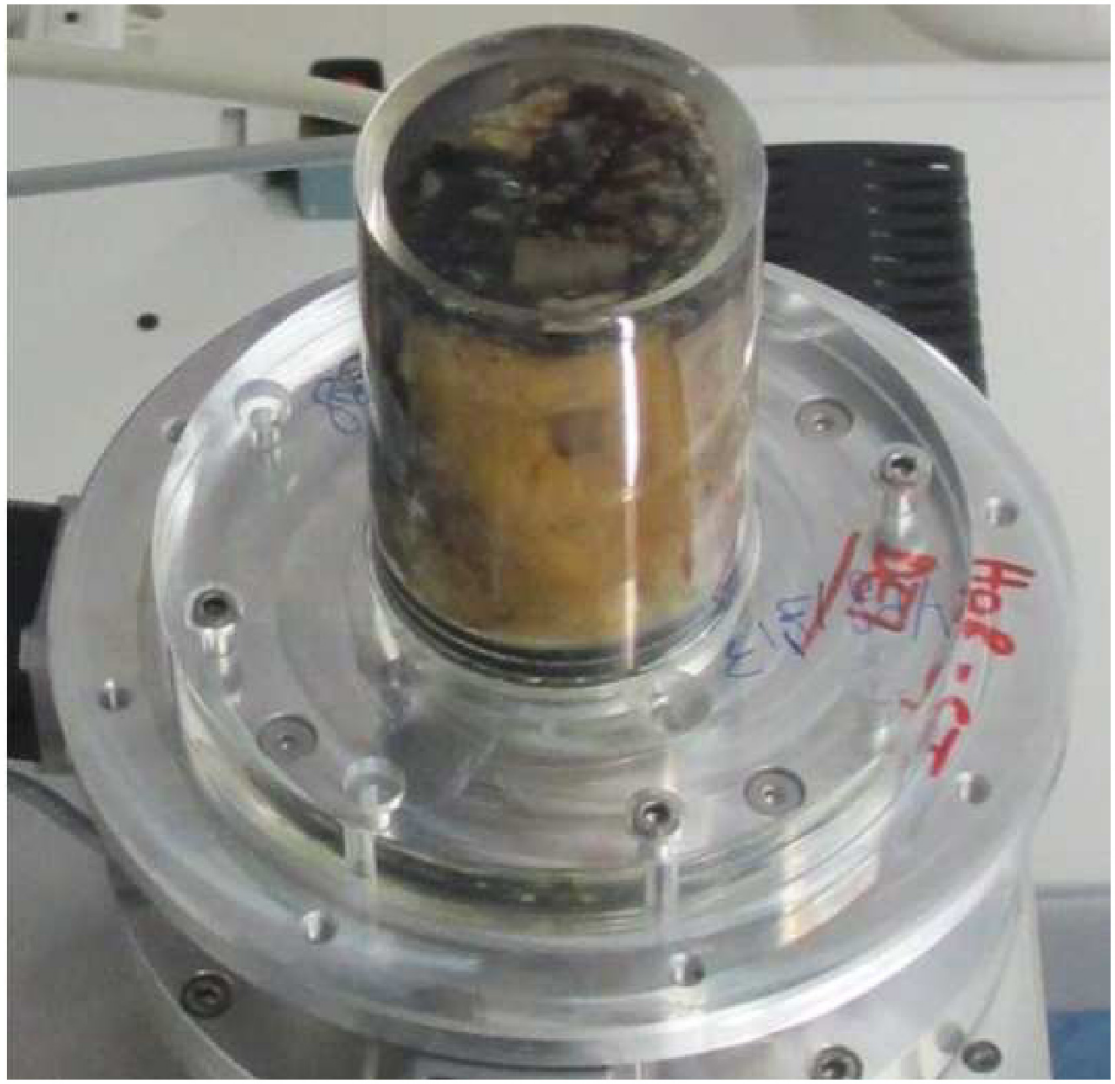
Sample-holder used for PC-CT imaging. Photograph of the sample-holder with the sample in it.

**Fig 2:**
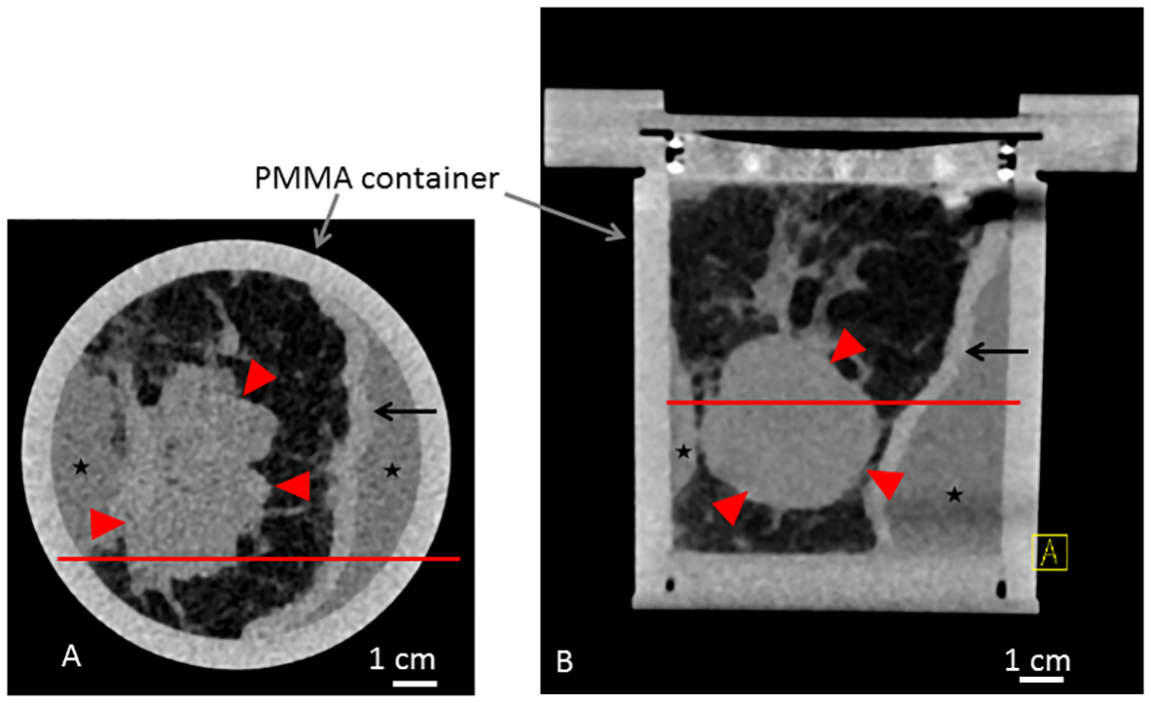
Clinical CT scan of sample A. Clinical CT scan of sample A in the sample holder with a standard clinical whole body CT scanner (Somatom Definition Flash, Siemens, Germany). Axial (A) and coronal (B) view. Red line in (A) and (B) reference for the respective imaging position. Asterisk indicating formaldehyde surrounding the sample. Red arrowheads indicating tumor. Arrows indicating skin.

**Fig 3:**
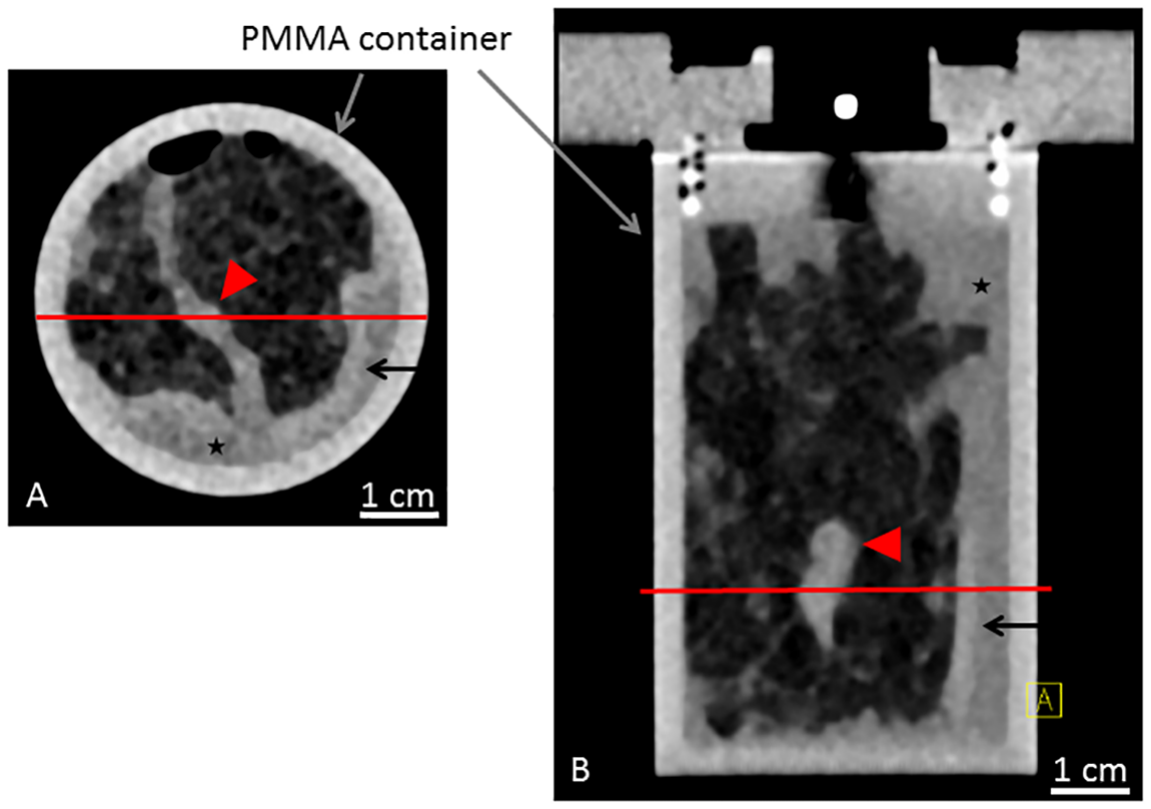
Clinical CT scan of sample B. Clinical CT scan of sample B in the sample holder with a standard clinical whole body CT scanner (Somatom Definition Flash, Siemens, Germany). Axial (A) and coronal (B) view. Red line in (A) and (B) reference for the respective imaging position. Asterisk indicating formaldehyde surrounding the sample. Red arrowheads indicating tumor. Arrows indicating skin.

Within one hour after surgery, the mastectomy samples (sample A and B) were fixated in a 4% formaldehyde-solution. Clinical standard histopathological workup was completed before the PC-CT imaging session. In short, the formaldehyde-fixated samples were cut into 5-mm slices. The slices remained attached to the intact skin enabling later recomposition. Macroscopically suspicious and representative tissue sections (max. 3.0 x 2.0 x 0.5 cm^3^) were resected for standard paraffin embedding and automatic staining. The samples were stored in a 4% formaldehyde-solution until imaging. For imaging purposes, the samples were manually recomposed and placed into a custom made PMMA cylindrical sample-holder shown in Fig 1. The remaining free airspace was filled with 4% formalin solution. The samples were 10 and 7 cm in diameter, respectively. The samples were scanned in a standard clinical whole body CT scanner (Somatom Definition Flash, Siemens, Germany) for a better overview and comparison with experimental data. The respective images of sample A and B are shown in Figs 2 and 3, respectively.

### Computed Tomography of the Index of Refraction: Imaging Principles and Description of the Setup

X-ray phase-contrast imaging techniques enable acquisition of images (projections), whose intensity is proportional to the spatial derivative of the phase delays experienced by X-rays upon propagation in the sample. The phase delays are related to the index of refraction δ of various features and tissues composing the sample. The 3D distribution of the refractive index inside the object can be reconstructed from a tomographic set of differential phase projections by means of the filtered backprojection (FBP) algorithm for transvers gradient projections [23]. Similar to the conventional absorption X-ray computed tomography (CT), where the composition and the morphology of the sample is determined based on difference in the linear attenuation coefficient expressed in Hounsfield units [24], the internal structure of the object can be also visualized using images of the refractive index δ. The advantage of reconstructing δ instead of the attenuation coefficient lies in the fact that the relative difference in the refractive indices between soft tissues is several times larger than the corresponding difference in the linear attenuation coefficient. For instance, at a photon energy of 50 keV, the refractive indices of adipose and glandular tissues differ by about 3%, which is twice the relative difference in the linear attenuation coefficient [25]. Moreover, as explained in [23], the filter used in FBP algorithm for gradient projections does not amplify the noise in a way the ordinary FBP filter does, therefore the quality of the reconstructed index of refraction images is intrinsically better than those based on the attenuation coefficient. Higher contrast of index of refraction images allows distinguishing minute variations in the breast tissue density, which is necessary for accurate visualization of the post-therapeutic effects.

**Fig 4:**
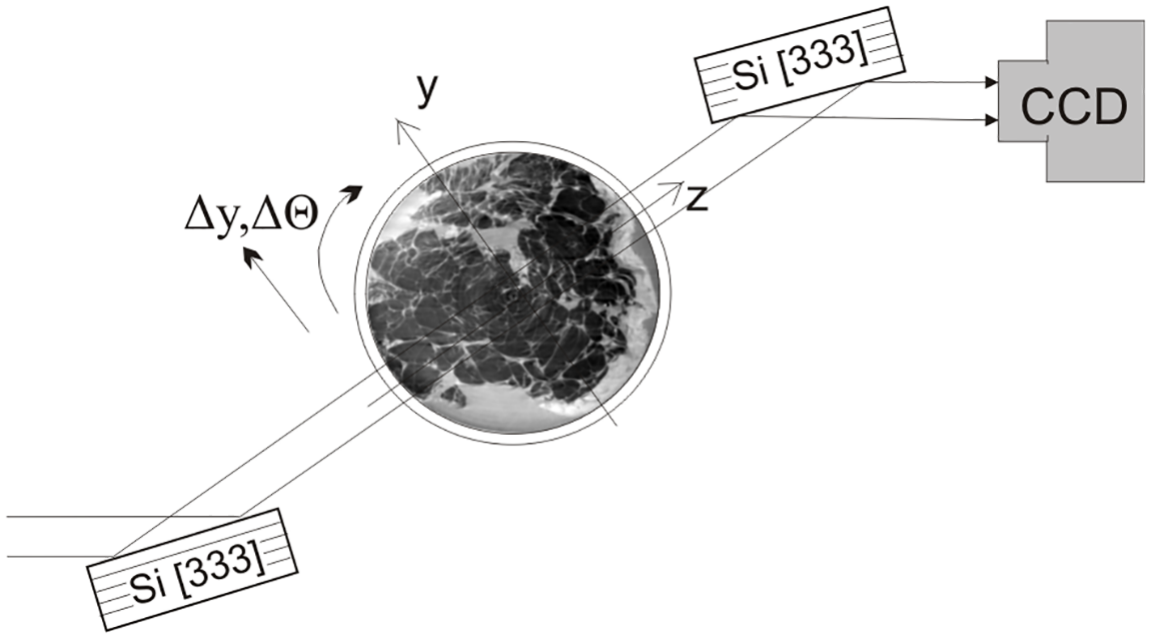
Basic principles of the ABI Method. Scheme of the imaging system used for the acquisition of phase-contrast tomographic data. A monochromatic X-ray beam impinges on the sample. After its interaction with matter the refracted beam is filtered by the analyzer crystal before reaching the detection system. The whole setup used in the study measures around 3 m.

In this work the PC-CT experiment was performed at the biomedical beamline (ID17) of the European Synchrotron Radiation Facility, Grenoble, France (ESRF). Analyzer-based crystal setup was used to obtain raw phase-contrast images. Fig 4 presents a sketch of the setup. X-ray beam was monochromatized and collimated upon reflection from a perfect Si crystal. Phase delays occurring in the beam after propagation through an object were converted to the image contrast by means of beam reflection from the second crystal, identical to the first one [see ref 20–22 for details]. A pair of raw phase-contrast images was used to obtain each of differential phase-contrast projections using the algorithm described in [26]. In total 1000 raw projections were obtained yielding 500 differential phase-contrast projections used for the index of refraction CT reconstruction. The acquisition time required to acquire the necessary data was about 1.5 hours per sample. The photon energy was 51 keV and a voxel size was 100 × 100 × 100 μm^3^. Detailed description of the setup, acquisition process, and data processing can be found in work [27].

The dose deposited into the tissues during the image acquisition was about 100 mGy. The dose values have been calculated using the method published in the work of Mittone et al. [28]. Such a large value is attributed to the low photon detection efficiency of the indirect imaging system, which is about 3% at the X-ray energy used in this study. The deposited dose and, correspondingly, the acquisition time can be reduced by a factor of forty if a single photon counting detector (characterized by high quantum efficiency here, for instance, assumed of 60%) and advanced (single set based) approaches for the extraction of the refraction information [29] are used. An additional gain can be obtained if low-dose CT algorithms requiring fewer angular projections with respect to standard filtered-back projection are applied [27, 30].

### Histological Workup

After completion of the PCI representative tissue sections were dehydrated in an ascending alcohol series before embedding in hot paraffin wax. Since the sample size exceeded standard size, the procedure was performed manually by enwrapping the samples in a blotting-paper and using non-standard slides (7.5 x 6.5 cm^2^). After solidification, the paraffin blocks were cut into 5 μm sections using a standard microtome and sections were stained with hematoxylin and eosin using standard protocols. Tumor diameters were calculated during the histopathological standard workup using standard rulers.

### Correlation between Histological Slices and ABI Data

The examination of the pathological signs in histology was used as the gold standard for the comparison with tomographic data. Images were evaluated referring to tumor size, tumor boundary, tumor sclerosis and microcalcifications according to the Sinn scale (0 = no effect, 1 = resorption and tumor sclerosis, 2 = minimal residual invasive tumor, 3 = only residual noninvasive tumor, 4 = no residual tumor detectable).The PC-CT images were interpreted by 2 radiologists experienced in clinical mammography in consensus. On the reconstructed PC-CT volume of the imaged breast, several slice orientations were investigated in order to find the best correlation with the produced histological cut. The best fitting slices were chosen and a visual co-registration between the different acquisition techniques was performed. The size calculations were performed by extrapolation from well-defined landmarks such as the diameter of the sample-holder.

## Results

**Fig 5:**
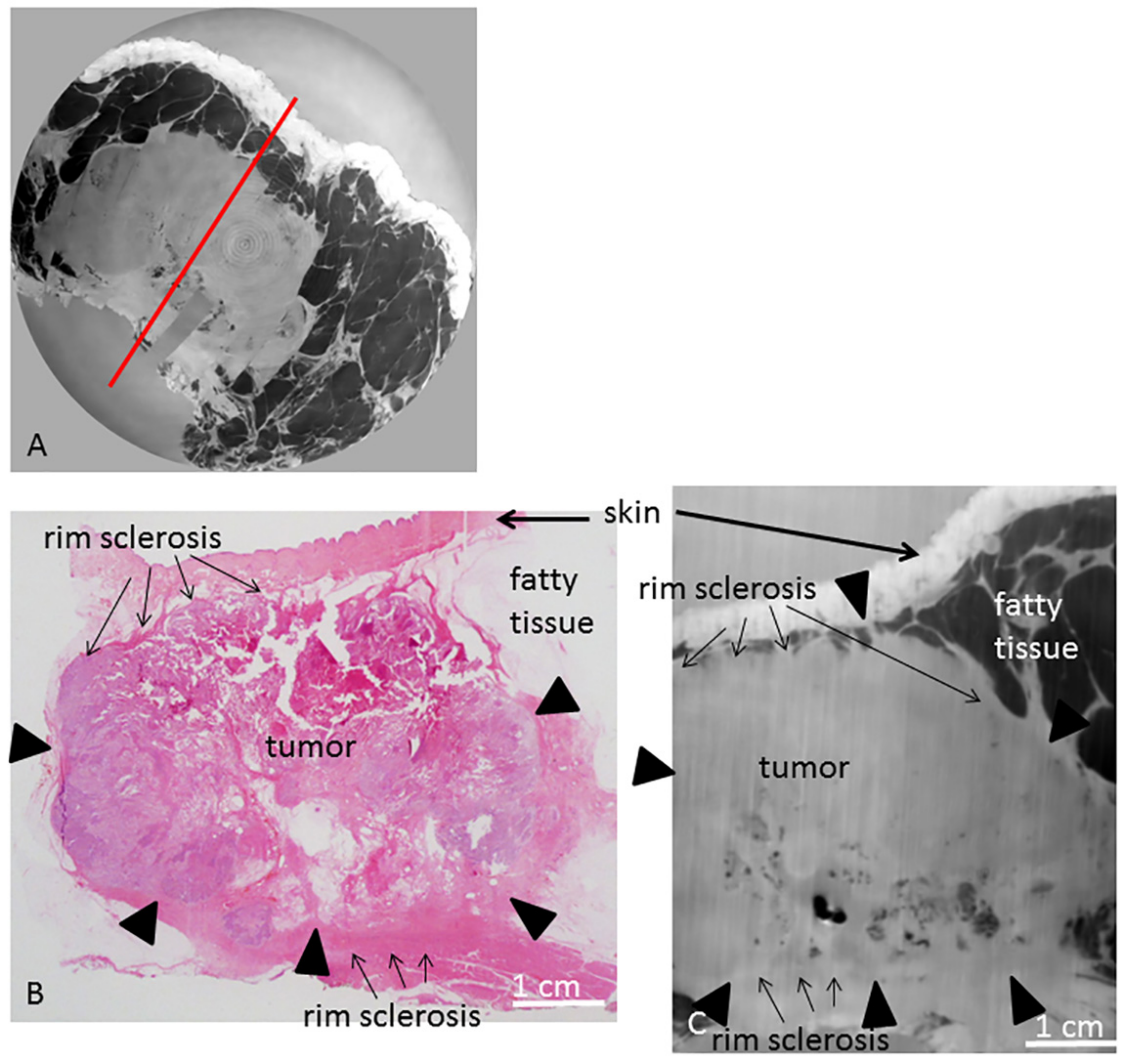
PC-CT and histology of sample A. PC-CT image of the whole sample volume in planar orientation (A), representative histological slice in hematoxlin eosin staining (B) and corresponding PC-CT slice (C). Tumor borders are indicated by arrowheads. Red line in (A) indicates cutting direction of (B) and (C). Fat arrows indicate skin; small arrows indicate rim sclerosis.

**Fig 6:**
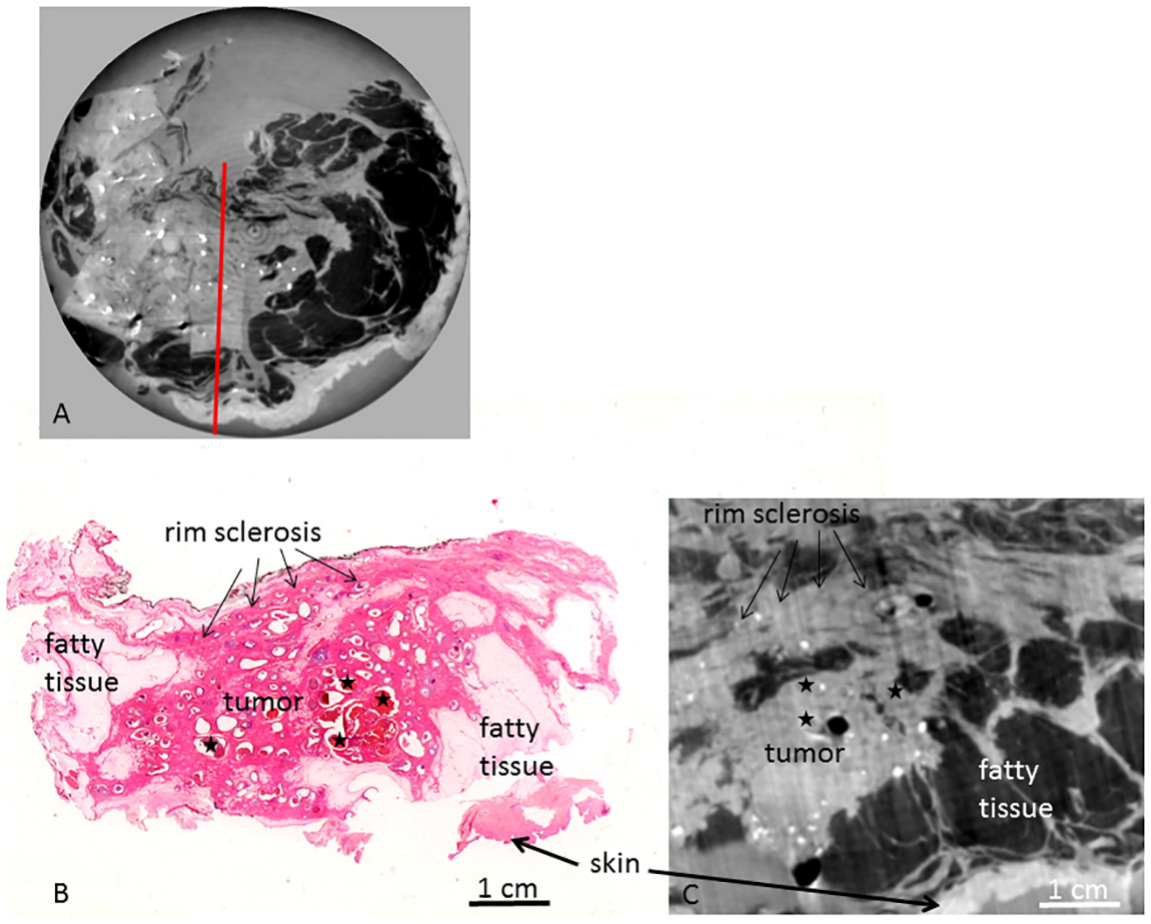
PC-CT and histology of sample B. PC-CT image of the whole sample volume in planar orientation (A), representative histological slice in hematoxlin eosin staining (B) and corresponding PC-CT slice (C). Red line in (A) indicates cutting direction of (B) and (C). Fat arrows indicate skin; small arrows indicate rim sclerosis. Asterisks indicate in situ carcinoma.

Information about patient A and B and sample A and B are shown in Table 1. Correlation of PC-CT data and histopathology was successfully completed. In both samples, the remaining tumor was visible in PC-CT in three dimensions. An overview of the PC-CT image data of sample A and B in planar projection is shown in Figs 5A and 6A respectively. It is possible to detect some artifacts in Figs 5A, 5C, 6A and 6C represented by rings and stripes. These artifacts are related mainly to small instabilities of the system during the acquisition.

**Table 1:** Characteristics of patient A and B: age, histological diagnosis and tumor type (NST = invasive carcinoma no special subtype; DCIS = ductal carcinoma in situ); classification of mammographic breast density according to the American College of Radiology (ACR); average glandular dose (AGD in mGy) of the preoperative in-vivo mammography in craniocaudal and mediolateral oblique projection (cc / mlo), dimensions and weight of the breast samples; tumor extensions according to histopathology; TNM classification; therapy response according to standard clinical imaging.

Patient	A	B
**Age** (years)	58	56
**Histological diagnosis**	NST, G3	Residual DCIS, G3
**ACR**	III	IV
**AGD (mGy)**	4.50 / 3.55	1.38 / 1.78
**Sample dimensions / weight**	190 x 185 x 45 mm^3^ / 1138 g	145 x 83 x 29 mm^3^ / 163 g
**Histological tumor diameter**	62 x 57 x 36 mm^3^	58 x 42 x 21 mm^3^
**TNM mastectomy specimen**	ypT3, ypNx	ypTis, ypN0
**Clinical therapy response**	progression suspected	Stable disease

In order to reduce these artifacts more advanced post processing methods can be applied during the reconstructions of the images.

### Sample A

**Fig 7:**
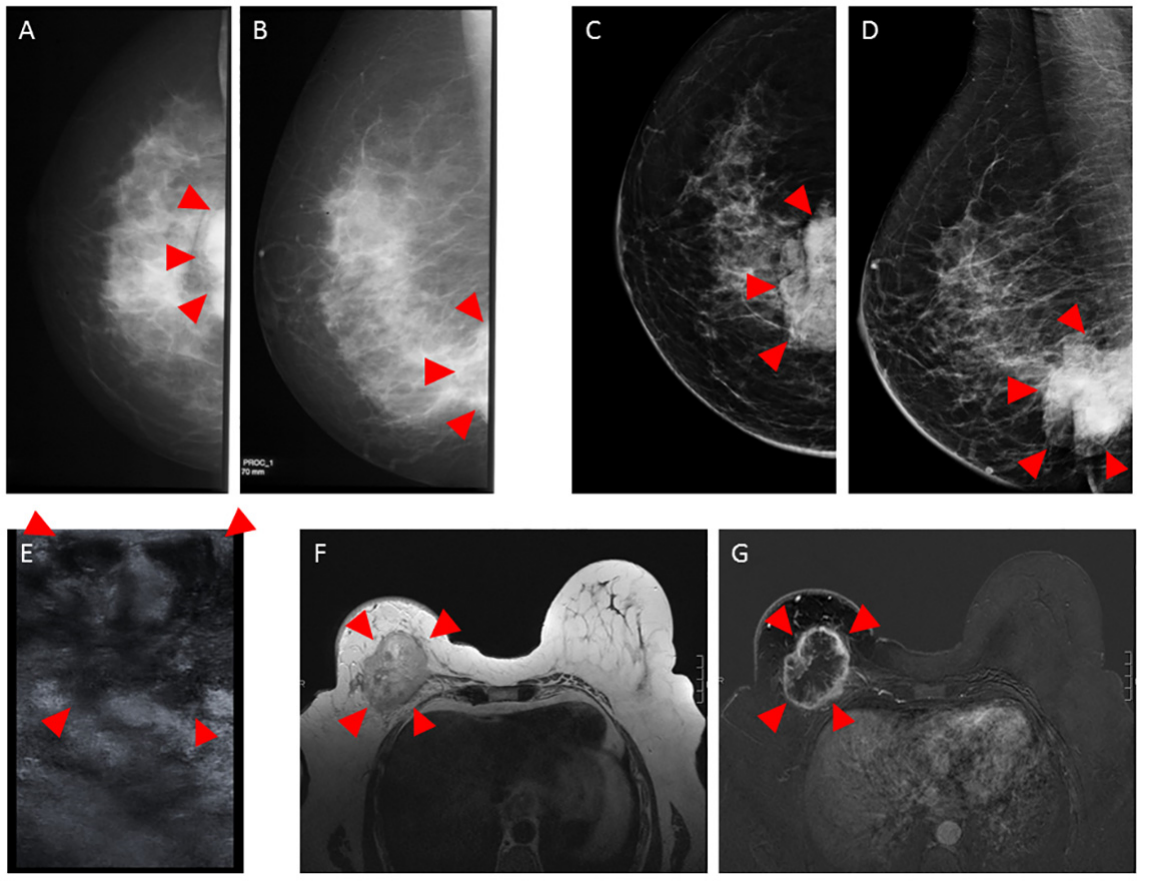
Clinical mammography, sonography and MRI of patient A. In-vivo mammography of patient A in craniocaudal (A and C) and mediolateral oblique (B and D) projection before (A and B) and after completion of NAC (C and D). Ultrasound before NAC (E). In-vivo MRI including contrast-enhanced T1 weighted gradient-echo sequence after manual injection of 30 ml gadopentetate dimeglumine (Magnevist ^®^ 0.5 mmol/ml) (F) and the corresponding first subtraction image after 2 min (G) in an axial view using a dedicated sensitivity-encoding enabled bilateral breast coil with a 1.5-Tesla system. The tumor is marked with arrowheads. Please note that the tumor is only partially imaged in conventional mammography due to the prepectoral position (A–D) and in Ultrasound (E) due to the extensive size.

Fig 7 shows the in-vivo mammography of patient A before (Fig 7A and 7B) and after (Fig 7C and 7D) completion of NAC. The mammograms only show parts of the immediately prepectoral carcinoma. Sonographically, the total tumor extension could not be measured due to the extensive tumor size (Fig 7E). As such, a comparison between pre- and post-therapeutic imaging was difficult. In conclusion, according to mammography and ultrasound the therapy response was questionable. The pre-therapeutic MRI of patient A is shown in Fig 7F and 7G and reveals the carcinoma as contrast-enhancing 8.0 x 7.2 x 6.2 cm^3^ lesion.

Macroscopic examination of sample A showed a partly whitish, partly yellow, knotty configured tumor with a diameter of 6.2 x 5.8 x 4.2 cm^3^.

Histology revealed a residual invasive carcinoma NST (no special subtype, formerly invasive ductal carcinoma) of 6.2 cm maximum diameter, in correlation with the pre-therapeutic MRI (maximum tumor diameter of 8.0 cm) reveals this a decrease of tumor size of approximately 2 cm. A representative hematoxylin-eosin-stained slice is shown in Fig 5B, showing adhering skin layers, fatty tissue and the tumor with fibrosis and necrosis. These tumor necrosis and fibrosis represent the post-therapeutic effects, altogether a poor, but visible response to NAC (regression grade 1 according to the Sinn classification = resorption and tumor sclerosis). The tumor infiltrates close to the epidermis. In the posterior parts, adhering muscle tissue can be differentiated.

Fig 5C shows the corresponding PC-CT slice of sample A. The epidermis corresponds to a zone of high contrast. Fatty tissue is dark in PC-CT. The carcinoma is clearly differentiable from skin and fatty tissue. The residual tumor has a compact, homogeneous appearance in a bright grey, enabling a differentiation from the adhering fibrous tissue, skin and fatty tissue as well as from the adhering layers of skeletal muscle. The boundaries of the tumor are well visible enabling a correct estimation of the post-therapeutic tumor size (6.2 x 6.1 x 4.2 cm^3^ in PC-CT versus 6.2 x 5.8 x 4.2 cm^3^ in histology). There are scattered brighter areas within the tumor and around the tumor, in correlation with histopathology conformable with sclerotic areas representing post-therapeutic tissue changes (regression grade 1 according to the Sinn classification = resorption and tumor sclerosis). PC-CT reveals that the tumor infiltrates close to the epidermis but shows a thin layer of fatty tissue (dark) between tumor and epidermis; this finding is in concordance with histopathology where the tumor infiltration is reported close to the epidermis without skin infiltration. The remaining breast parenchyma is displaced by the extensive tumor. Strands of fibrous tissue interspersing the adjacent fatty tissue are differentiable from the carcinoma in terms of different grey levels.

### Sample B

**Fig 8:**
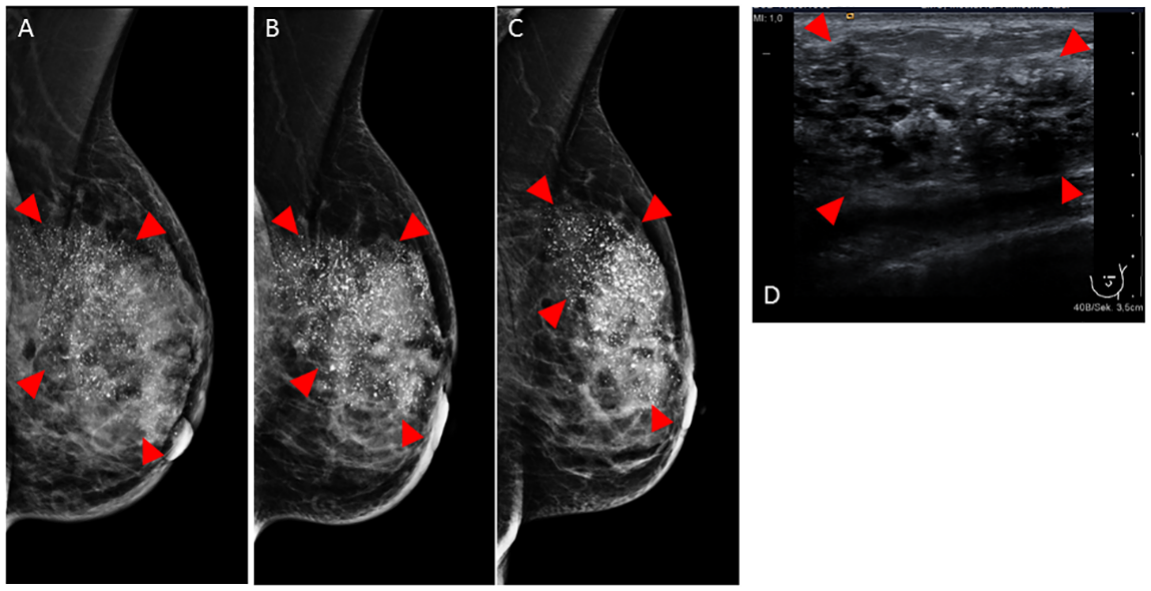
Clinical mammography and sonography of patient B. In-vivo mammography in mediolateral oblique projection before (A), after 4 cycles NAC (B) and after completion of NAC (C). Ultrasound before NAC (D). Tumor indicated by arrowheads. Please note the excessive calcifications (white spots in A–D) within the tumor. The exact tumor borders are not visible in mammography and sonography.

Fig 8 shows the conventional in-vivo mammography of patient B before (A), after 4 cycles (B), and at the end (C) of NAC. Mammography shows extensive calcifications within the left breast. No compact tumor component is visible so that the exact tumor extension cannot be measured. During the course of NAC, the diffuse mammographic hyperdensity of the breast parenchyma appears to resolve a bit but there are no measurable changes in the image features so that the response to NAC cannot be evaluated. Ultrasonography (Fig 8D) before NAC is not able to show a compact tumor or the tumor margins, so tumor size evaluation by ultrasound was not feasible.

Macroscopic examination of sample B showed a whitish tumor with a diameter of 6 x 4 x 2 cm with an irregular shape. The tumor was fibrous and compact with a microcystic surface.

Histology revealed a residual ductal in situ carcinoma (DCIS) of 5.8 cm maximum diameter with extensive comedo necroses and microcalcifications. Fig 6B shows a representative histological slice of sample B. Histopathology shows a centrally located, large area of tumor sclerosis, inflammation and dystrophic stromal calcifications following NAC. The tumor area clearly shows post-therapeutic tissue changes: extensively sclerotic breast tissue filled with ectatic ducts, the latter containing atypical epithelial cells corresponding to residual in situ carcinoma. No residual invasive carcinoma is visible. The response to NAC was classified as grade 3 according to the Sinn classification (only residual noninvasive tumor).

PC-CT (Fig 6C) enables an excellent visualization of histological structures: PC-CT shows bright spots as a correlate of the multiple microcalcifications within the DCIS. The in situ components (dark spots) are clearly visualized within intact, dilated ducts. The complete DCIS area is embedded within bright areas of sclerotic tissue changes which are compatible with areas of tumor regression (post-therapeutic changes). In contrast to sample A, no compact growing invasive tumor component is seen. Summing up the post-therapeutic tissue changes seen with PCI (tumor sclerosis, no compact invasive tumor visible) and correlate them with histopathology, we can confirm the results of histopathology (Grade 3 according to the Sinn classification). The skin is clearly differentiable from fatty tissue. Adhering muscle tissue is visualized in a light grey color.

## Discussion and Conclusion

A series of studies performed on tumor-bearing breast samples indicate that reconstructed hard X-ray index of refraction CT images provide an excellent soft-tissue contrast and an improved visualization of fine structures including collagen strands, microcalcifications and strands of lobular carcinoma [20, 31]. Sztrokay et al. showed that PC-CT but not absorption-contrast CT is able to visualize the intraductal tumor-component of ductal carcinoma in situ [32]. With increasing use of NAC, a correct prediction of response to NAC is of high relevance. However, the inter-observer variability of clinical examination, mammography and ultrasonography in the assessment of response to NAC is high. Currently, the definitive evaluation of response to NAC relies on postoperative histopathology as the gold-standard. The sensitivity of mammography in the detection of NAC-induced regressive variations is limited and the post-therapeutic tumor size tends to be overestimated. The application of the PCI method has been proven to allow a better observation of the breast architecture, as well as spiculated evaginations and microcalcifications in comparison with conventional mammography. In this study, we demonstrate for the first time that ABI-CT allows the visualization of NAC-induced regressive tissue changes in whole mastectomy samples, especially for diffuse growing tumors. We measured two mastectomy samples applying PC-CT. Both samples contained an invasive carcinoma and both patients had undergone NAC. The PC-CT images showed an area of compact growing tumor in sample A representing a large residual carcinoma with extensive tumor necrosis and sclerosis. In sample B, no compact tumor was visible in PC-CT but a large area of sclerosis with diffuse dilated ducts containing microcalcifications and intraductal tumor components could be clearly differentiated from “normal” breast tissue. The pathological work-up showed in sample A a Sinn score 1, in sample B a Sinn score 3. Hence, in contrast to conventional mammography and ultrasound, PCI was able to show the post-therapeutic tissue changes, especially in sample B (in mammography and ultrasound no measurable tumor was seen) and so PCI monitors the therapy response. In the future, PC-CT may thus be used as a new method for visualizing the response of breast tumors to NAC and could become a tool for visualizing and thereby bordering diffuse tumor areas which cannot be detected with the conventional mammography and ultrasound (f.e. diffuse DCIS, lobular invasive tumors). Pending significant dose reduction, PC-CT might serve as an additional in-vivo imaging tool in cases where conventional imaging methods fail to monitor therapy response. While our study was performed using a non -optimized setup and with dose levels exceeding the recommended dose for mammography by far, PC-CT of whole breasts has been performed at clinically acceptable doses [18]. In these cases, PC-CT could combine the advantages of histology and three-dimensional imaging as it is provided by MRI but at a resolution formerly restricted to histopathology. In an ex-vivo setting, PC-CT might assist histopathological diagnostics. Even if the resolution is inferior to histopathology, it provides a three-dimensional dataset and might help to identify diagnostically relevant features within large samples.

To our knowledge, this is the first study assessing the value of PC-CT as a method for therapy-response monitoring. The acquired PC-CT data provide correct visualization of histopathological structures in excellent correlation with histopathology and reflect the response to NAC correctly. In this work the ABI technique has been used for the investigation, however other differential PCI techniques can be employed as well. Some of these, while based on a similar contrast mechanism and providing similar sensitivity, are more suitable to be used with X-rays produced by other sources than synchrotron radiation machine. A very important step for a possible future application of the PCI techniques in clinics is linked to the development of compact sources and detectors. A combination of these new compact sources, advanced detectors and PCI techniques less stringent in terms of beam property may provide a new powerful diagnostic tool for routine examinations.

Limitations of our study include the small number of samples and the necessity of data acquisition after formalin fixation with a consequent flattening of the image contrast. The effect of formalin fixation on breast cancer specimens complicates the comparison with preoperative in-vivo imaging. However, recent studies suggest that the tumor diameter does not substantially differ between non-fixated and fixated specimens [33, 34]. The authors acknowledge that a deduction from the two selected cases to a general patient population is not justified. However, our data encourage further evaluation of PC-CT imaging in the evaluation of therapy response of breast cancer.
